# Professional Relationship Between Physicians and Journalists in Bangladesh: Web-Based Cross-Sectional Study

**DOI:** 10.2196/44116

**Published:** 2023-07-10

**Authors:** Md Aminul Islam, Md Golam Rabbani, Zamilur Rahaman, Taufique Joarder

**Affiliations:** 1 Department of Media Studies and Journalism University of Liberal Arts Bangladesh Dhaka Bangladesh; 2 Public Health Foundation Bangladesh Dhaka Bangladesh; 3 Health Economics Unit Health Services Division Ministry of Health and Family Welfare Dhaka Bangladesh; 4 Monno Medical College Monno City Manikganj, Dhaka Bangladesh; 5 SingHealth Duke-NUS Global Health Institute Singapore Singapore

**Keywords:** medicine, media, physician, journalist, communication, health policy, Bangladesh

## Abstract

**Background:**

A health care system is intertwined with multiple stakeholders, including government institutions, pharmaceutical companies, patients, hospitals and clinics, health care professionals, health researchers and scientific medical experts, patients and consumer organizations, and media organizations. Physicians and journalists are the key actors who play a significant role in making health care services and health information accessible to the people of a country.

**Objective:**

The aim of this study was to explore the tensions and alliances between physicians and journalists in Bangladesh, along with identifying strategies that could potentially improve the often contentious relationship and quality of medical journalism.

**Methods:**

We conducted a web-based cross-sectional survey using the snowball sampling technique from September 2021 to March 2022. Adult Bangladeshi citizens belonging to the two selected professional groups (physicians and journalists), who understood the survey content, and agreed to participate in the survey were considered eligible for inclusion in the study. Both descriptive and logistic regression analyses, including the Mann-Whitney *U* test and Wilcoxon signed-rank test, were performed to determine the differences between groups concerning selected perception-related variables, and the associations of perceptions about lack of trust in each other’s knowledge, skills, and professional integrity with background characteristics.

**Results:**

A total of 419 participants completed the survey, including 219 physicians and 200 journalists. Among physicians, 53.4% (117/219) reported lower trust toward journalists’ professional domain and expertise, whereas 43.5% (87/200) of journalists had lower trust toward physicians’ professional domain and expertise. In terms of perception about not having respect for each other, the median value for the physicians was 5 (strongly agree), whereas it was only 3 (agree) for the journalists. We also found that male physicians (adjusted odds ratio [AOR] 0.45, compared with female physicians) and medical officers (AOR 0.30, compared with specialists) had significantly higher odds of lacking trust in journalists’ knowledge, skills, and professional integrity. When rating the statement “Regular professional interaction between journalists and doctors may improve the relationship between the professional groups,” most physicians (186/219, 84.9%) chose “neither agree nor disagree,” whereas most journalists (106/200, 53.0%) stated that they “slightly agree.”

**Conclusions:**

Both physicians and journalists in Bangladesh have negative perceptions of each other’s professions. However, physicians have a more negative perception of journalists than journalists have of the physicians. Strategies such as a legal framework to identify medical-legal issues in reporting, constructive discussion, professional interaction, and capacity-building training programs may significantly improve the relationship between physicians and journalists.

## Introduction

### Background

A health care system is intertwined with multiple stakeholders, including government institutions, pharmaceutical companies, patients, hospitals and clinics, health care professionals, health researchers and scientific medical experts, patients and consumer organizations, and media organizations. Physicians and journalists are the key actors who play a significant role in making health care services and health information accessible to the people of a country. Media translate complex health issues, health policies, scientific medical innovations, and research updates for the public, patients, practitioners, and policy makers. People’s health-related behaviors [1]; beliefs, attitudes, and actions [2]; and perceptions of the quality of health care services are influenced and shaped by media content. Therefore, media play a crucial role in framing public health debates [3] and the public health policy process [4,5].

Nevertheless, health and media are closely connected in many other ways. Briggs and Hallin [6] examined the relationship between media and medicine. They argued that news coverage of health issues plays a fundamental role in constructing wider cultural understandings of health and disease. Moreover, Stroobant et al [7] argued that health news is coproduced by health and media professionals. However, authors of previous studies explored the relationship between health and media through the lens of certain professional domains. In most cases, they adopted either the media perspective [6] or a health care perspective [8] exclusively. Thus, it is evident that the current discourse on the relationship is divided into two lines of thought. On the one hand, media professionals often argue that doctors do not know how to express themselves in a way that nonmedical professionals can understand, they do not appreciate journalists’ skills and/or act as if they are superior or omnipotent, they try to take over/dominate the journalistic process, and they have a personal agenda when collaborating with the media. On the other hand, health professionals claim that the media cannot be trusted as journalists often report health-related issues in a biased, sensational, and inaccurate way; they do not understand the complexity of health care and the health care business; and are often responsible for breaches of confidentiality or privacy, or choose to misquote health professionals [9].

It is well established that media can play an influential role in promoting health behavior [10-14], the use of health care services [12,15-18], building people’s trust in the health care system [19-23], and advancing health literacy [24,25] in a country. A cordial relationship between physicians and journalists is therefore crucial for improved health care delivery and the public health of a country. However, physicians and medical scientists often argue that the media frequently negatively portray the health sector and health care professionals and use exaggerated headlines that lower the quality of medical messages in the media. On the other side, media professionals argue that health care professionals do not cooperate with them in communicating medical messages properly. However, work on incorporating both perspectives is scant. In particular, there is no research on the topic in the context of Bangladesh.

Against this backdrop, the aim of this study was to explore the professional relationship between physicians and journalists in Bangladesh. The goal is to identify strategies that could potentially improve this often contentious relationship by characterizing the tensions and alliances between medicine and the media in Bangladesh and the quality of medical journalism in the country.

### Theoretical Framework

The aim of this study was to examine the professional relationship between physicians and journalists through the theoretical lenses of biocommunicability [26], biomediatization [6], and boundary-work theory [27]. The notion of biocommunicability refers to the ways biomedical knowledge is created, circulated, and received [6,26]. According to this concept, media and medicine are two distinct but intensively interactional entities. By contrast, the biomediatization concept implies that biomedicine and the media are not two separate entities but are rather deeply intertwined. Both medicine and media contribute to the production of medical knowledge, the practice of medicine, and public health [6,7]. How media construct and communicate health knowledge affects perceptions of particular diseases, public health policies, clinic practices, and public reactions because the media frame health news through multiple social, economic, cultural, political, and moral lenses [6,28].

The concept of boundary work was first introduced by Thomas F Gieryn in 1983, which refers to an ideological demarcation between scientific and nonscientific fields [27]. Gieryn argued that various professional groups and occupations construct social boundaries that distinguish some intellectual activities. They put up such boundaries in arguing for their power, authority, control, credibility, expertise, prestige, and material resources. Moreover, they play rhetorical games for their objectivity and the need for autonomy. As an analytical instrument, this concept is particularly useful in understanding the professional relationship between physicians and journalists [29-32]. Thus, to understand the tensions and alliances between medicine and the media in Bangladesh, we sought to identify the prejudices physicians and journalists have against one another toward finding possible solutions that could improve the mutual relationship and the quality of medical journalism in the country.

Medical journalism refers to journalistic communication of issues related to health, medicine, and the health care system. In essence, medical journalism is another form of journalistic writing about science [33,34], representing an art and craft of telling complex stories on structural, institutional, political, financial, and ethical issues in health, medicine, and health care [35] in a way that enables a lay person to easily understand these issues.

## Methods

### Study Design, Setting, and Participants

This was a cross-sectional online survey. To achieve the objectives of this study, a quantitative approach was adopted. As the aim was to capture the perceptions of both physicians and journalists working in Bangladesh, two separate surveys were created, each comprising common variables and participant-specific variables. The surveys were conducted between September 2021 and March 2022. The call for participation was made on social media and by email.

### Recruitment Procedure

We collected data through an anonymous web-based survey using social media platforms and email. Two semistructured questionnaires (for physicians and journalists, respectively) were designed using the Google survey tool (Google Forms). The generated link was shared with physicians and journalists identified through the snowball technique. The link was also shared with study participants via social media groups. Through the link, the study participants could access the relevant questionnaire as well as read a brief description of the study, with its objectives, implications, and data management guidelines. Informed consent was obtained from all participants through the same web link. After providing consent, a participant was able to access the remainder of the questionnaire, which also included the contact addresses of the research team and an Ethical Review Committee member, allowing them to reach out for further queries or clarification regarding the study. The participants were not required to provide any personal or identifiable information on the questionnaires. To maintain data quality, the research team checked the data regularly to determine whether there were any inconsistencies.

We collected data from professional physicians/registered physicians/clinical practitioners such as senior consultants, junior consultants, teaching professionals of medical colleges, and residential medical officers/medical officers or equivalent who work in primary, secondary, and tertiary government hospitals and medical colleges, as well as private clinics and private medical colleges across the country. In addition, any registered journalist working in print, television, or online news platforms was considered eligible for the journalist survey. All Bangladeshi citizens aged 20 years or above that belonged to the two selected professional groups, understood the survey content, and agreed to participate in the survey were considered eligible for inclusion in the study. A total of 419 of 528 participants completed the survey, with a response rate of 79.35%, including 219 physicians and 200 journalists.

### Study Instruments

We developed the physician and journalist perception questionnaires following the existing literature, after which we customized these items to the Bangladeshi context and translated them into Bangla. The questionnaires included sociodemographic and profession-related questions. The physician perception questionnaire included questions on their experience of professional interactions with journalists, perceptions of the impact of media on the health care sector, perceptions of the importance of a good relationship between medicine and media, prejudice about media and journalists, and suggestions for improving the relationship. The journalist perception questionnaire comprised questions on their perceptions about health care professionals, knowledge about health and medical reporting, the experience of interactions with physicians, perceptions about the importance of a good relationship between medicine and media, prejudice about physicians and health care professionals, and suggestions for improving the relationship. The questionnaires are provided in Multimedia Appendix 1.

### Statistical Analysis

We performed both descriptive and inferential statistical analyses. The descriptive analysis focused on frequencies (n) and percentages, and the Mann-Whitney *U* test and Wilcoxon signed-rank test were performed to determine the differences between groups (physicians and journalists) concerning selected perception-related variables. Internal consistency of the perception variables between the two groups was tested using the Cronbach α coefficient. The Cronbach α for the common perception (7 items) variables between the two groups was .776, indicating a satisfactory internal consistency level [36,37]. Moreover, we performed a reliability test for the physician perception–related variables toward journalists (12 items) and the journalist perception–related variables (9 items) toward physicians. The Cronbach α score for the physician and journalist groups was .893 and .814, respectively, indicating a satisfactory internal consistency level [36,37]. Multiple ordered logistic regression analyses jointly considering all the explanatory variables were performed to assess the association between the background characteristics of the two study groups with a common perception variable, formed based on the rating of the survey item “Not having trust in each other’s knowledge, skills, and professional integrity” on a 5-point Likert scale anchored at 1=“strongly disagree” and 5=“strongly agree.” A *P* value <.05 was considered statistically significant. We analyzed the data using Stata SE, version 15.0 (StataCorp LLC).

### Ethics Considerations

The Ethical Review Committee (PHFBD-ERC: 12/2020) of the Public Health Foundation, Bangladesh approved our study protocol, procedures, consent statement, and study tools. All respondents were informed in Bengali about their rights related to their voluntary participation in the study. Participants who gave consent to willingly participate in the survey would click the “Continue” button and would then be directed to complete the self-administered questionnaire. Respondents were assured of the anonymity of the data they provided.

## Results

### Background Characteristics of Physicians and Journalists

A total of 219 physicians and 200 journalists residing in Bangladesh completed the questionnaire. Among the physicians, the mean age was 38.86 (SD 9.94) years and the mean duration of professional experience was 12.68 (SD 8.56) years. Nearly three-quarters of the participants identified as male; 31.05% worked as specialists and 12.79% as consultants. In addition, 43.38% of the participants were currently based in Dhaka, the capital city of Bangladesh (Table 1). Among the journalists, the mean age was 33.97 (SD 9.24) years and the mean duration of professional experience was 9.85 (SD 8.19) years; nearly three-quarters were male. In addition, 48.5% worked as a reporter and 61.5% were currently working in Dhaka (Table 1).

**Table 1:** Background characteristics of the study participants (N=419).

Variables		Physicians (n=219)	Journalists (n=200)
Age (years), mean (SD)		38.86 (9.94)	33.97 (9.24)
Years of professional experience, mean (SD)		12.68 (8.56)	9.85 (8.19)
**Sex, n (%)**			
	Male	159 (72.6)	149 (74.5)
	Female	59 (26.9)	48 (24.0)
	Prefer not to say	1 (0.5)	3 (1.5)
**Physicians’ professional title, n (%)**			
	Medical officer	111 (50.7)	N/A^a^
	Junior consultant	12 (5.5)	N/A
	Consultant	28 (12.8)	N/A
	Specialist	68 (31.1)	N/A
**Journalists’ professional title, n (%)**			
	Correspondent	N/A	53 (26.5)
	Reporter	N/A	97 (48.5)
	News editor	N/A	42 (21.0)
	Others (eg, anchor, media manager)	N/A	8 (4.0)
**Current working place, n (%)**			
	Capital city (Dhaka)	95 (43.4)	123 (61.5)
	Other divisional city	43 (19.6)	24 (12.0)
	District	56 (25.6)	35 (17.5)
	Upazila^b^ and Union^c^	25 (11.4)	18 (9.0)

^a^N/A: not applicable.

^b^An administrative division in Bangladesh, functioning as a subunit of a district.

^c^The smallest rural administrative and local government unit in Bangladesh.

### Physicians’ Perceptions Toward Journalists and Journalists’ Perceptions Toward Physicians

The perceptions of the two professional groups toward each other were assessed based on seven common domains (Table 2). Both physicians and journalists ranked their perceptions toward each other using a 5-point Likert scale, with 1 denoting “strongly disagree” or having a very negative perception and 5 indicating “strongly agree” or having a very positive perception. The complete data are provided in Multimedia Appendix 2.

Six out of seven variables regarding physicians’ perception toward journalists and journalists’ perception toward physicians were statistically significant at the 5% level (*P*<.05). Among physicians, 53.4% reported that they had lower trust toward journalists’ professional domain and expertise, whereas among journalists, 43.5% of participants had lower trust toward physicians’ professional domain and expertise. Moreover, 56.2% of physicians were of the view that journalists have a “very low” level of professionalism, whereas only 32.5% of the journalists perceived physicians to have a “very low” level of professionalism.

When rating the statement “Journalists often do not have respect for physicians as a professional group,” 50.7% of physicians “strongly agreed.” In contrast, only 10.5% of journalists “strongly agreed” with the same statement regarding the physicians. Similarly, 51.6% of physicians “strongly agreed” with the statement “Journalists do not have trust in the knowledge, skills, and professional integrity of physicians,” whereas only 4.5% of journalists “strongly agreed” when rating the statement “Physicians do not have trust in media and journalists in the country.”

When rating the statement “Journalists tend to believe that they are superior to physicians as a professional group,” 86.8% of physicians “strongly agreed.” In contrast, only 19.0% of journalists “strongly agreed” with the corresponding statement regarding the physicians. In addition, 82.2% of physicians “strongly agreed” with the statement “When reporting on the health sector, journalists often tend to serve the purpose of vested business interests, not representing the truth.” In contrast, only 1.0% of journalists “strongly agreed” with the statement “Physicians often prescribe medicine or tests to ensure the interests of pharmaceutical companies.” Finally, 89.5% of physicians, as opposed to 18.5% of journalists, expressed their strong agreement with the statement “The relationship between physicians and journalists is not good.”

**Table 2:** Physicians’ perceptions toward journalists and journalists’ perceptions toward physicians in Bangladesh.

Question	Physicians (n=219), n (%)						Journalists (n=200), n (%)						*P* value^a^
	1^b^	2^c^	3^d^	4^e^	5^f^	1		2	3	4	5		
Trust toward each other’s professional domain and expertise	117 (53.4)	34 (15.5)	57 (26.0)	5 (2.3)	6 (2.7)	87 (43.5)		70 (35.0)	39 (19.5)	3 (1.5)	1 (0.5)	.72	
Perception about each other’s professionalism	123 (56.2)	54 (24.7)	38 (17.4)	2 (0.9)	2 (0.9)	65 (32.5)		77 (38.5)	47 (23.5)	7 (3.5)	4 (2.0)	<.001	
Perception about not having respect for each other	1 (0.5)	31 (14.2)	21 (9.6)	55 (25.1)	111 (50.7)	7 (3.5)		47 (23.5)	49 (24.5)	76 (38.0)	21 (10.5)	<.001	
Perception about not having trust in each other’s knowledge, skills, and professional integrity	0 (0)	31 (14.2)	20 (9.1)	55 (25.1)	113 (51.6)	4 (2.0)		35 (17.5)	49 (24.5)	103 (51.5)	9 (4.5)	<.001	
Perception toward each other’s superiority complex	0 (0)	4 (1.8)	3 (1.4)	22 (10.1)	190 (86.8)	7 (3.5)		15 (7.5)	17 (8.5)	123 (61.5)	38 (19.0)	<.001	
Belief toward each other about serving the purpose of vested interests	0 (0)	1 (0.5)	10 (4.6)	28 (12.8)	180 (82.2)	2 (1.0)		15 (7.5)	28 (14.0)	104 (52.0)	51 (25.5)	<.001	
Overall relationship is not good	0 (0)	0 (0)	2 (0.9)	21 (9.6)	196 (89.5)	1 (0.5)		19 (9.5)	27 (13.5)	116 (58.0)	37 (18.5)	<.001	

^a^Mann-Whitney *U* test.

^b^Very low/strongly disagree.

^c^Slightly low/slightly disagree.

^d^Neither low/agree nor high/disagree.

^e^Slightly high/slightly agree.

^f^Very high/strongly agree.

### Median Values of the 5-Point Likert Scale Ratings for Different Perception Aspects

**Figure 1:**
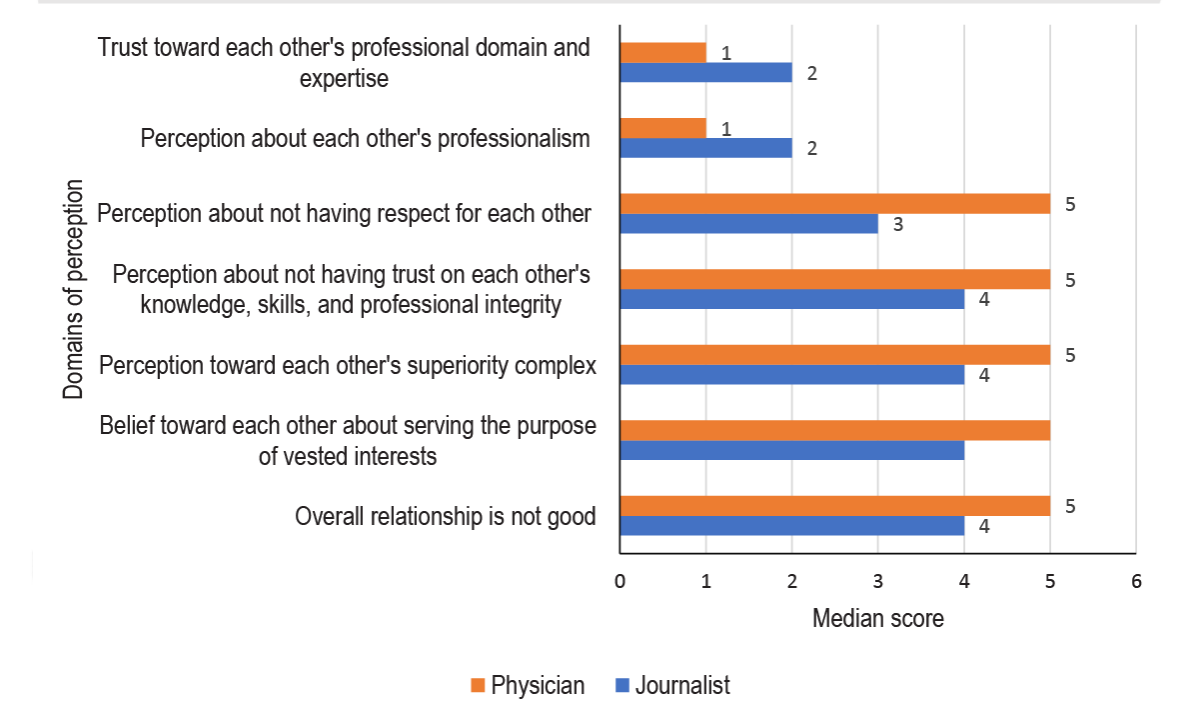
Median values of the 5-point Likert scale on different domains of perceptions (1=strongly disagree and 5=strongly agree) for journalists and physicians.

In terms of the statements “Trust toward each other’s professional domain and expertise” and “Perception about each other’s professionalism,” the median value for the physicians was 1, whereas it was 2 for the journalists. In terms of “Perception about not having respect for each other,” the median value for the physicians was 5, whereas it was 3 for the journalists. In terms of other variables such as “Not having trust in each other’s knowledge, skills, and professional integrity”; “Perception toward each other’s superiority complex”; “Beliefs toward each other about serving the purpose of vested interests”; and “Overall relationship is not good,” the median value for the physicians was 5, whereas it was 4 for the journalists (Figure 1).

### Factors Associated With Lack of Trust in Each Other’s Knowledge, Skills, and Professional Integrity

The ordered logistic regression analysis showed that several factors—sex and the professional title for physicians, and age, designation, and current workplace for journalists—were significantly associated with the lack of trust in each other’s knowledge, skills, and professional integrity (Table 3).

In terms of the background characteristics of physicians, male physicians had significantly higher odds of lacking trust in journalists’ knowledge, skills, and professional integrity compared to their female counterparts. In terms of professional title, medical officers had significantly higher odds of lacking trust in journalists’ knowledge, skills, and professional integrity compared to the specialists.

On the other side, in terms of the background characteristics of journalists, a 1-year increase in journalist age increased the odds of lacking trust in physicians’ knowledge, skills, and professional integrity by 0.14. The reporters and news editors had higher odds of lacking trust in physicians’ knowledge, skills, and professional integrity compared to the reference category, correspondents. In terms of current workplace, journalists from other city corporations and from the district level had higher odds of lacking trust in physicians’ knowledge, skills, and professional integrity compared with their reference category, journalists from Dhaka.

**Table 3:** Association of perceptions about not having trust in each other’s knowledge, skills, and professional integrity with background characteristics of physicians and journalists.

Variables			Physicians (n=219)^a^				Journalists (n=200)^b^		
			AOR^c^ (95% CI)		*P* value		AOR (95% CI)		*P* value
Age (years)			1.1 (1-1.21)		.06		1.14 (1.07-1.22)		<.001
Years of professional experience			0.91 (0.82-1.02)		.10		0.93 (0.87-1.00)		.06
**Sex**									
	Male (reference)	N/A^d^		N/A		N/A		N/A	
	Female	0.45 (0.25-0.81)		.008		1.88 (0.96-3.67)		.07	
	Prefer not to say	0 (0-0)		.99		4.58 (0.44-47.85)		.20	
**Physicians’ professional title**									
	Medical officer	N/A		N/A		N/A		N/A	
	Junior consultant	0.35 (0.11-1.15)		.09		N/A		N/A	
	Consultant	0.43 (0.17-1.12)		.09		N/A		N/A	
	Specialist	0.3 (0.13-0.71)		.006		N/A		N/A	
**Journalists’ professional title**									
	Correspondent	N/A		N/A		N/A		N/A	
	Reporter	N/A		N/A		3.19 (1.42-7.15)		.005	
	News editor	N/A		N/A		3.34 (1.39-8.06)		.007	
	Others (eg, anchor, media manager)	N/A		N/A		3.44 (0.75-15.76)		.11	
**Current working place**									
	Capital city (Dhaka)	N/A		N/A		N/A		N/A	
	Others divisional city	0.59 (0.29-1.19)		.14		4.26 (1.67-10.88)		.002	
	District	0.73 (0.38-1.41)		.35		3.56 (1.51-8.38)		.004	
	Upazila^e^ and below	0.55 (0.21-1.41)		.21		1.2 (0.43-3.36)		.73	

^a^Model parameters: Likelihood ratio (*χ^2^*_10_)=24.62, *P*=.006; Pseudo *R^2^*=0.047.

^b^Model parameters: Likelihood ratio (*χ^2^*_10_)=39.54, *P*=.001; Pseudo *R^2^*=0.081.

^c^AOR: adjusted odds ratio.

^d^N/A: not applicable.

^e^An administrative division in Bangladesh, functioning as a subunit of a district.

### Physicians’ Perceptions Toward Journalists

Table 4 presents the physicians’ perceptions of journalists in Bangladesh, measured through 12 variables, 6 of which were statistically significant (*P*<.05). A few of the significant findings are summarized below.

Most physicians “strongly agreed” with the statements “Journalists often prepare news stories on their own first and then talk to physicians,” “Journalists often present health and medical information in a sensational way,” “Journalists often use the term ‘wrong treatment’ without considering the context or details,” and “Journalists often tend to publish news stories on health care professionals and health care services without adequate verification.”

**Table 4:** Physicians’ perceptions toward journalists (N=219).

Variable	Strongly disagree, n (%)	Slightly disagree, n (%)	Neither agree nor disagree, n (%)	Slightly agree, n (%)	Strongly agree, n (%)	*P* value^a^
Journalists often write and publish news on the health sector without having adequate knowledge about it	0 (0)	9 (4.1)	14 (6.4)	33 (15.1)	163 (74.4)	.26
Journalists often write and publish news stories based on their preconceived ideas	0 (0)	2 (0.9)	28 (12.8)	34 (15.5)	155 (70.8)	.44
Journalists often prepare news stories on their own first and then talk to physicians	1 (0.5)	2 (0.9)	36 (16.4)	30 (13.7)	150 (68.5)	<.001
Journalists often do not try to understand the real situation or the underlying meaning of a medical situation; rather, they are more interested in what they want to know	1 (0.5)	3 (1.4)	5 (2.3)	28 (12.8)	182 (83.1)	.63
In most cases, journalists present a distorted picture of health professionals and health care services	0 (0)	4 (1.8)	4 (1.8)	27 (12.3)	184 (84.0)	.48
The media always publish biased information on the health sector and health professionals	0 (0)	2 (0.9)	5 (2.3)	25 (11.4)	187 (85.4)	.13
Journalists often present health and medical information in a sensational way	1 (0.5)	1 (0.5)	2 (0.9)	25 (11.4)	190 (86.8)	.002
Journalists often use the term “wrong treatment” without considering the context or details	0 (0)	0 (0)	1 (0.5)	17 (7.8)	201 (91.8)	.004
In most cases, journalists incorrectly quote physicians or health care professionals in their news stories	0 (0)	0 (0)	6 (2.7)	23 (10.5)	190 (86.8)	.32
Journalists often tend to publish news stories on health care professionals and health care services without adequate verification	0 (0)	1 (0.5)	2 (0.9)	22 (10.1)	194 (88.6)	<.001
I am afraid of talking to journalists as they do not know how to ask questions objectively/neutrally	1 (0.5)	7 (3.2)	8 (3.7)	22 (10.1)	181 (82.7)	.01
Journalists tend to believe that most physicians are not qualified and inhumane	0 (0)	1 (0.5)	8 (3.7)	22 (10.1)	188 (85.8)	<.001

^a^Wilcoxon signed-rank test.

### Journalists’ Perceptions Toward Physicians

Table 5 presents the journalists’ perceptions of physicians in Bangladesh, measured through 9 variables, 7 of which were statistically significant (*P*<.05). In particular, most journalists “slightly agreed” with the statements “During an interview or in case of communicating information relevant to a news story, most physicians tend not to give enough time to journalists”; “While talking to media, physicians use jargon and difficult terms that are not understandable for ordinary persons”; “During an interview, physicians often try to dominate over journalists”; and “Physicians often try to avoid media and journalists as a result of their professional supremacy attitude.”

**Table 5:** Journalists’ perceptions toward physicians (N=200).

Variables	Strongly disagree, n (%)	Slightly disagree, n (%)	Neither agree nor disagree, n (%)	Slightly agree, n (%)	Strongly agree, n (%)	*P* value^a^
Physicians often tend to believe that journalists do not have adequate knowledge about the country’s health care system	5 (2.5)	24 (12.0)	35 (17.5)	119 (59.5)	17 (8.5)	.22
When contacting for any information relevant to a story, physicians often pretend that they are too busy	3 (1.5)	26 (13.0)	36 (18.0)	108 (54.0)	27 (13.5)	.02
Physicians often seem not to be confident while appearing in media or talking to journalists	1 (0.5)	27 (13.5)	43 (21.5)	120 (60.0)	9 (4.5)	.70
Most physicians are not skilled in giving an interview or talking to journalists	4 (2.0)	41 (20.5)	36 (18.0)	100 (50.0)	19 (9.5)	.02
During an interview or in case of communicating information relevant to a news story, most physicians tend to not give enough time to journalists	4 (2.0)	19 (9.5)	35 (17.5)	128 (64.0)	14 (7.0)	.002
While talking to the media, physicians use jargon and difficult terms that are not understandable to ordinary people	2 (1.0)	17 (8.5)	32 (16.0)	109 (54.5)	40 (20.0)	.01
Physicians often do not feel the need to present medical information in a simple, straightforward manner	3 (1.5)	24 (12.0)	33 (16.5)	116 (58.0)	24 (12.0)	<.001
During an interview, physicians often try to dominate journalists	18 (9.0)	25 (12.5)	48 (24.0)	96 (48.0)	13 (6.5)	<.001
Physicians often try to avoid media and journalists as a result of their professional supremacy attitude	3 (1.5)	13 (6.5)	34 (17.0)	124 (62.0)	26 (13.0)	.01

^a^Wilcoxon signed-rank test.

### The Way Forward From the Perspectives of Physicians and Journalists

**Figure 2:**
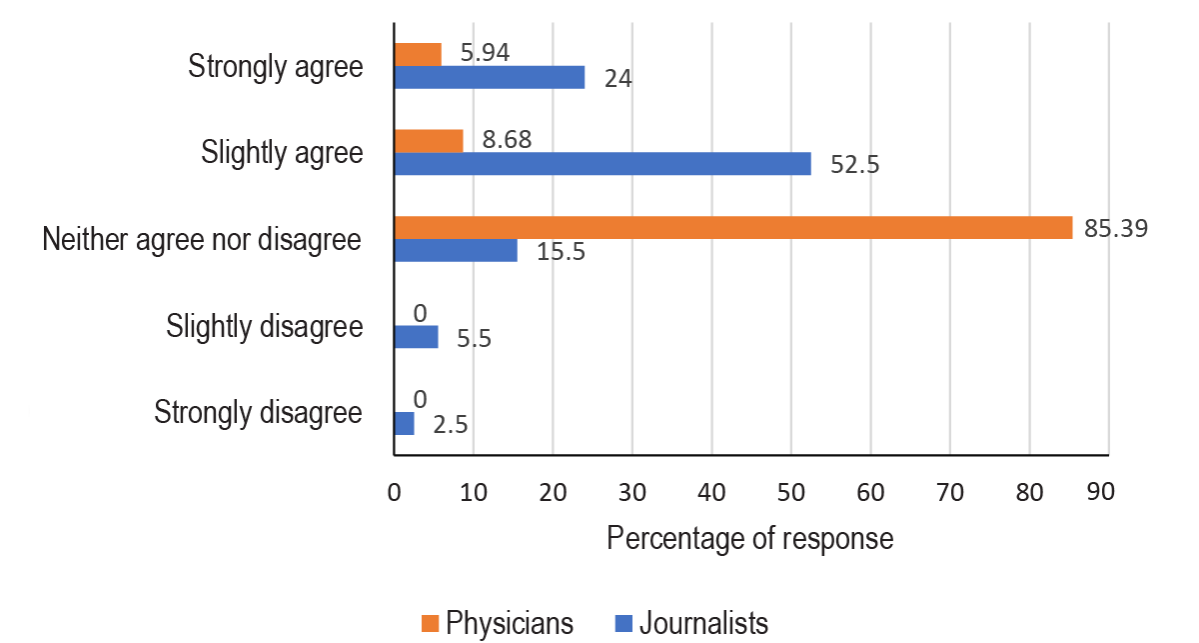
Responses to the statement "More interaction between physicians and journalists may improve the relationship between the professional groups.".

When rating the statement “Regular professional interaction between journalists and doctors may improve the relationship between the professional groups,” most physicians (85%) chose “neither agree nor disagree,” whereas most journalists (53%) stated that they “slightly agree” (Figure 2).

**Figure 3:**
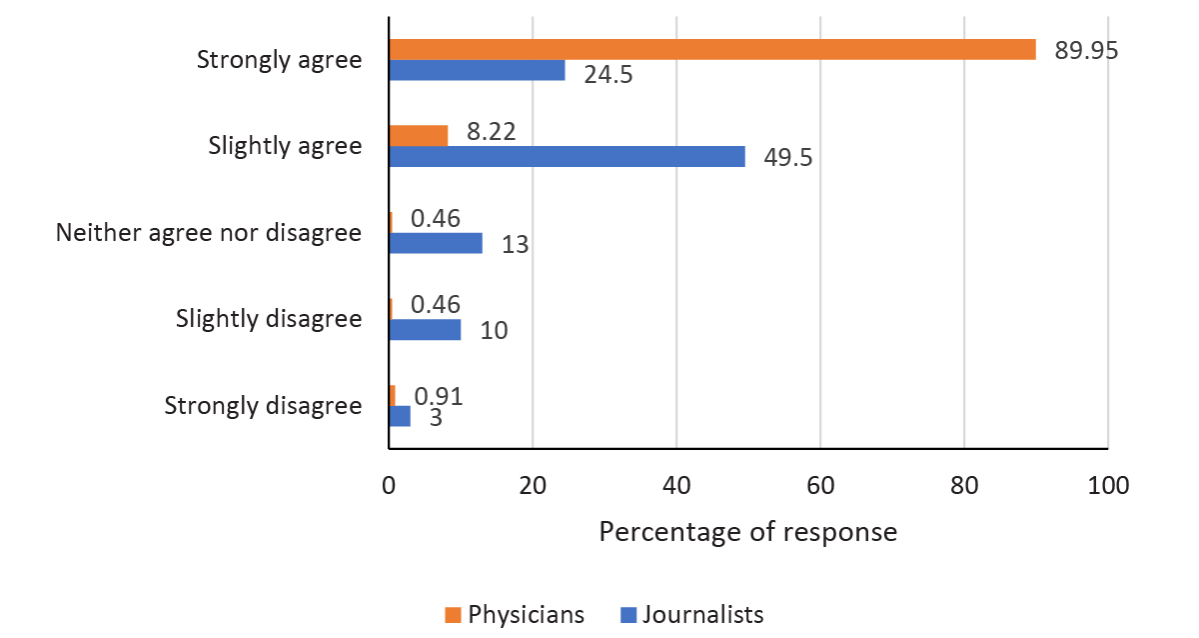
Responses to the statement "Necessary training may improve the relationship between the professional groups.".

When rating the statement “Necessary training may improve the relationship between the professional groups,” most physicians (90%) chose “strongly agree,” whereas most journalists (50%) opted for “slightly agree” (Figure 3).

## Discussion

### Principal Findings

From the overall results, it is evident that both physicians and journalists in Bangladesh have a negative perception of each other. However, physicians have a more negative perception of the journalists than the journalists have of the physicians. We also found that the attitude of male and junior physicians toward the journalists is more negative compared with the attitudes of other physician groups.

The negative attitude of the professional groups has been exemplified by the variables such as trust toward the opposite professional group; perception regarding professionalism and not having respect for each other; and not having trust in each other’s knowledge, skills, and professional integrity. The high level of negative perceptions of physicians may be attributed to several factors. For example, in medical college, students are given an impression of superiority over other professional groups. This phenomenon has been observed by Zaman [38] in the Bangladeshi context. There is a historical link between traditional enmity and distrust toward each other [39-41]. It is also possible that a lack of knowledge, understanding, and training about medical and health issues among journalists often leads to misreporting on these issues, which harms the professional credentials of physicians. The issue of misreporting was echoed in the opinions of physicians that participated in this study, as they believe that journalists often present health and medical information in a sensational way, often use the term “wrong treatment” without considering the context or details, and tend to publish news stories on health care professionals and health care services without adequate verification. A similar perception of physicians toward journalists was observed by Ahlmén-Laiho et al [42,43] in the context of Finland. However, these authors argued that journalists’ experience of collaboration with physicians was positive. There are several cases of journalists portraying incidents of patients dying at a hospital or a clinic as an outcome of the ignorance and negligence of physicians, which could be a reason for the enmity from the physicians’ perspective. The negative portrayal of individual physicians, and the health care system as a whole, may affect public trust in physicians and the health care system [44-46]. In another study, Ahlmén-Laiho et al [42] found that physicians often do not trust health information published or broadcasted in news media.

Our results indicate that physicians in Bangladesh are not comfortable talking to journalists, while the journalists are skeptical of physicians’ communication skills. This might be due to the lack of communication skills among physicians, as the medical training curriculum does not adequately include behavioral science and communication skills, especially on how to face media. This failure often leads to negative perceptions of physicians among journalists, which is reflected in their news reporting. The negative perception may stem from the low standard of general journalistic practices, particularly in reporting on medical and health issues. Journalists often work on tight deadlines and write overstated headlines while covering health issues, compromising the relationship between professional groups [47]. However, Leask et al [1] argued that the relationship could be improved through physicians’ increased awareness of journalists’ work culture and daily routines, being available when journalists request an interview or any piece of information for their news stories, providing them with necessary resources, and building relationships with specialist health reporters.

Our results show that the attitude of junior physicians is more negative toward journalists compared to that of senior physicians, aligning with previous findings in the United Kingdom [48]. This attitude might be attributed to the communication skills and experience of facing journalists in professional encounters. Perhaps junior physicians might have less developed skills in facing media. However, journalists may also be less critical of senior physicians that have greater experience in facing media and journalists. We also found that male physicians and medical officers are more negative toward journalists compared to specialists. The same explanation may apply to this finding.

Overall, our results show that the relationship between two professional groups—journalists and physicians—is not good, concurring with the results obtained in previous studies [49]. The differences in work cultures often lead to negative perceptions. For example, in their study on 600 medical experts in 21 countries, Larsson and colleagues [47] found that the nature of journalists’ work, short deadlines, writing populist headlines, their choice of topics or angles in news stories, and their level of medical knowledge are some of the barriers to overcome to improve the quality of medical reporting. However, the negative attitudes of the two professional groups toward each other are harmful to the quality of care and may undermine their professional motivation.

Empirical evidence indicates that the lack of adequate communication between the two professional groups is one of the key barriers to the dissemination of public health information in a country. An improved relationship between the two professional groups to enhance their understanding of each other’s work culture is thus required. Medical colleges should incorporate issues related to communication skills with both patients and news media in the medical training of their graduates. On the other side, journalism schools should incorporate medical and health issues in their curricula so that future journalists can be equipped with the necessary medical knowledge. Moreover, the government should formulate a legal system to address medical negligence to ensure evidence-based representation of health issues.

### Limitations

This study has some limitations. First, the participants of this study were only selected among users of social media platforms and email. Thus, there may be response bias, and only those concerned more about medicine and media among physicians and journalists may have participated in the study. Second, the study is based on a nonrepresentative sample size. Therefore, the results may not be generalizable to other physicians and journalists who are not social media or email users. Third, this study was exploratory, which did not allow for making any causal inferences.

### Strengths

Despite these limitations, the study’s strength lies in the fact that it provides the first scientific evidence on the relationship between physicians and journalists in Bangladesh, to our best knowledge. Another key strength of the study is that it has considered the perspectives of both physicians and journalists to reveal the professional relationship between these two groups.

Further qualitative research is nonetheless still needed to understand this phenomenon in greater depth. Qualitative formative research will help design interventions to enrich professional skill sets, responsiveness, and communication strategies to improve and maintain a sustainable healthy relationship.

### Conclusion

Both physicians and journalists in Bangladesh have negative perceptions of each other. The perception of physicians toward journalists appears to be more negative than the perception of journalists toward physicians. Our findings suggest that several strategies could be adopted to improve the existing unhealthy relationship between these two important professional groups in Bangladesh and other contexts. First, a legal framework is needed to identify medical-legal issues in reporting. Moreover, policy makers should take initiatives related to constructive discussion, professional interaction, and capacity-building training programs, which may significantly improve the relationship between physicians and journalists.

## Appendix

Multimedia Appendix 1 Study questionnaire.

Multimedia Appendix 2 Study data file.

## Data Availability

All data generated or analyzed during this study are included in this published article and its supplementary information files.
