# Supplementary material for: Professional Relationship Between Physicians and Journalists in Bangladesh: Web-Based Cross-Sectional Study
Source: Interact J Med Res. 2023 Jul 10;12:e44116. doi: 10.2196/44116 (PMC10366956; doi:10.2196/44116)
Supplement: Multimedia Appendix 1 [file ijmr_v12i1e44116_app1.docx]

**Study Title:** Professional Relationship between Physicians and Journalists in Bangladesh: A Web-Based Cross-sectional Study

[Journalist perspective]

**Section A: Socio-demographic information of respondents**

1. Age (in complete years): …………………. Years

2. What is your gender identity?

1. Male

2. Female

3. Prefer not to say

3. What is your highest educational attainment?

1. Postgraduate or above

2.Graduate/ MBBS

3. HSC or below

4. What is your professional title?

1. Special correspondent

2. Senior correspondent

3. Reporter

4. Junior reporter

5. Senior sub-editor

6. Sub-editor

7. Others, specify

5. Where is your current workplace?

1. Capital city (Dhaka)
2. Any other city corporation

2. District level

3. Upazila level

4. Union level

6. The length of your experience as professional journalist: ……………………. Years

**Section B:** Journalist perception toward physician in Bangladesh

| **Variables** | **Journalists’ perception** | | | | | |
| --- | --- | --- | --- | --- | --- | --- |
|  | Very high/ Strongly agree | Very low/ Strongly disagree | Slightly low/ Slightly disagree | Neither low/ agree nor high/ disagree | Slightly high/ Slightly agree | Very high/ Strongly agree |
| Trust towards each other's professional domain and expertise |  |  |  |  |  |  |
| Perception about each other's professionalism |  |  |  |  |  |  |
| Perception about not having respect for each other |  |  |  |  |  |  |
| Perception about not having trust in each other's knowledge, skills, and professional integrity |  |  |  |  |  |  |
| Perception towards each other's superiority complex |  |  |  |  |  |  |
| Believe towards each other about serving the purpose of vested interests |  |  |  |  |  |  |
| Overall relationship is not good |  |  |  |  |  |  |

**Section C:** Journalists’ perception towards Physicians

| **Variables** | **Strongly disagree** | **Slightly disagree** | **Neither agree nor disagree** | **Slightly agree** | **Strongly agree** |
| --- | --- | --- | --- | --- | --- |
|  | **n (%)** | **n (%)** | **n (%)** | **n (%)** | **n (%)** |
| Physicians often tend to believe that journalists do not have adequate knowledge about the country's healthcare system |  |  |  |  |  |
| When contacting for any information relevant to a story, physicians often show business unreasonably |  |  |  |  |  |
| Physicians often seem not to be confident while appearing in media or talking to journalists |  |  |  |  |  |
| Most physicians are not skilled in giving an interview or talking to journalists |  |  |  |  |  |
| During an interview or in case of communicating information relevant to a news story, most physicians tend to not give enough time to journalists |  |  |  |  |  |
| While talking to the media, physicians use jargons and difficult terms that are not understandable to ordinary persons |  |  |  |  |  |
| Physicians often do not feel the need to present medical information in simple, straightforward |  |  |  |  |  |
| During an interview, physicians often try to dominate journalists |  |  |  |  |  |
| Physicians often try to avoid media and journalists as a result of their professional supremacy attitude |  |  |  |  |  |

**Thank you!**

**Study Title:** Professional Relationship between Physicians and Journalists in Bangladesh: A Web-Based Cross-sectional Study

[Physicians’ perspective]

**Section A: Socio-demographic information of respondents**

1. Your age: …………………. Years

2. Your gender identity:

1. Male

2. Female

3. Not willing to mention

3. Your highest-level educational attainment is:

1.Above postgraduate

2. Postgraduate

3. MBBS/ equivalent

4. Your professional title is:

1. Medical Officer/ Equivalent

2. Junior Consultant/ Equivalent

3. Consultant / Equivalent

4. Specialist/ Equivalent

5. Others

4. The length of your experience as a physician is: ……………………. Year

6. Your current workplace is located in:

1. Capital city (Dhaka)
2. Any other city corporation

2. District level

3. Upazila level

4. Union level

**Section B:** Physician perception toward journalist in Bangladesh

| **Variables** | **Physicians’ perception** | | | | |
| --- | --- | --- | --- | --- | --- |
|  | Very low/ Strongly disagree | Slightly low/ Slightly disagree | Neither low/ agree nor high/ disagree | Slightly high/ Slightly agree | Very high/ Strongly agree |
| Trust towards each other's professional domain and expertise |  |  |  |  |  |
| Perception about each other's professionalism |  |  |  |  |  |
| Perception about not having respect for each other |  |  |  |  |  |
| Perception about not having trust in each other's knowledge, skills, and professional integrity |  |  |  |  |  |
| Perception towards each other's superiority complex |  |  |  |  |  |
| Believe towards each other about serving the purpose of vested interests |  |  |  |  |  |
| Overall relationship is not good |  |  |  |  |  |

**Section C:** Physicians’ perception towards journalists

| **Variable** | **Strongly disagree** | **Slightly disagree** | **Neither agree nor disagree** | **Slightly agree** | **Strongly agree** |
| --- | --- | --- | --- | --- | --- |
| Journalists often write and publish news on the health sector without having adequate knowledge about it |  |  |  |  |  |
| Journalists often write and publish news stories based on their preconceived ideas |  |  |  |  |  |
| Journalists often prepare news stories on their own first and then talk to physicians |  |  |  |  |  |
| Journalists often do not try to understand the real situation or the underlying meaning of a medical situation; rather they are more interested in what they want to know |  |  |  |  |  |
| In most cases, journalists present a distorted picture of health professionals and healthcare services. |  |  |  |  |  |
| The media always publishes biased information on the health sector and health professionals |  |  |  |  |  |
| Journalists often present health and medical information in a sensational way |  |  |  |  |  |
| Journalists often use the term 'wrong treatment' without considering the context or details |  |  |  |  |  |
| In most cases, journalists incorrectly quote physicians or healthcare professionals in their news stories |  |  |  |  |  |
| Journalists often tend to publish news stories on healthcare professionals and healthcare services without adequate verification |  |  |  |  |  |
| I am afraid of talking to journalists as they do not know how to ask questions objectively/neutrally |  |  |  |  |  |
| Journalists tend to believe that most physicians are not qualified and inhumane |  |  |  |  |  |
| Regular professional interaction between journalists and doctors may improve the relationship between the professional group |  |  |  |  |  |
| Providing journalist with necessary training about healthcare and clinical process could improvement the relationship |  |  |  |  |  |

**Thank You**
